# Correlation between serum levels of microRNA-21 and inflammatory factors in patients with chronic heart failure

**DOI:** 10.1097/MD.0000000000030596

**Published:** 2022-09-23

**Authors:** Weiwei Li, Yanan Li, Feng Jiang, Huan Liu

**Affiliations:** a Emergency Department, Second Hospital of Lanzhou University, Lanzhou, China; b Aab Cardiovascular Research Institute, Department of Medicine, University of Rochester School of Medicine and Dentistry, Rochester, NY, USA.

**Keywords:** chronic heart failure, inflammation, microRNA, myocardial remodeling

## Abstract

As the leading cause of hospitalization and mortality worldwide, heart failure (HF) has caused significant burden on both individuals and the whole society. Thus, increasing knowledge about the phytopathology of HF is in demand for both diagnosis and treatment. Previous studies have shown that both microRNA 21 (miRNA-21) and inflammatory factors are closely related to the development of cardiac fibrosis, hypertrophy, and HF. However, whether there is any crosstalk between the 2 has not been examined. The current study evaluated the correlation between serum levels of miRNA-21 and critical inflammatory factors during the progress of chronic heart failure (CHF), providing new insights in understanding the physiopathology of CHF and identifying CHF biomarkers. In the presented study, serum level of miR-21, cardiac neurohormone, and critical inflammatory factors were measured and compared on 120 (67 male/53 female) CHF patients and 100 (58 male/42 female) health people with non-failing hearts. Echocardiography was also conducted to assess the severity of CHF. Correlations between different factors were calculated and tested for statistical significance. From our results, CHF patients showed significantly decreased serum levels of miR-21 while increased levels of inflammatory factors and cardiac neurohormone (*P* < .05). Levels of miR-21 negatively correlate with cardiac function while positively correlates with myocardial remodeling (*P* < .05). Levels of miR-21 negatively correlate with inflammation in CHF (*P* < .05). These findings indicate the potential crosstalk between serum miR-21 and inflammation during CHF progression, suggesting the potential of miR-21 in CHF diagnosis, severity indication, and treatment.

## 1. Introduction

Heart failure (HF) is the end stage of numerous cardiac muscle disorders, which develops secondary to left ventricular systolic and diastolic dysfunctions.^[1,2]^ It is the leading cause of hospitalization and mortality worldwide, with steadily increasing prevalence and incidence despite the significant advancements in the medical therapies preventing or treating HF.^[3]^ Besides, even though advanced medical care has increased the survival rate of patients after their first initial cardiac insult, the patients always suffer repeated and prolonged hospitalization with >50% mortality within 5 years.^[4]^ Given the staggering burden HF exerts on society, there is an ever-growing demand for improvements in both early diagnosis and treatment of HF, which may be achieved through improving our understanding of the mechanisms behind the pathogenesis of HF. Traditionally, diagnosis of HF depends on patients’ medical history, clinical symptoms, and physical examination. Risk factors for HF, such as high blood pressure, coronary artery disease, or diabetes, are also considered.^[5]^ These diagnostic methods require extensive information from both the patients’ and doctors’ experience and may not be accurate when accessing the severity of chronic heart failure (CHF). Thus, rapid diagnosis and an indication of CHF severity are in demand, which may be achieved through biomarkers.

The term HF is usually used to refer to CHF, which, unlike acute HF, is prevalent in the aged population and cause prolonged and repeated symptom.^[6,7]^ CHF can be classified as either HF with reduced ejection fraction (systolic HF) or HF with preserved ejection fraction (diastolic HF) based on whether reduced left ventricular ejection fraction (LVEF <40%) is observed.^[8]^ Both types contribute to ~50% of the CHF incidence and share similarities in symptoms and pathogenesis.^[9,10]^ Despite the different types and diverse initial causes of CHF, inflammation is always underlying the pathogenesis, especially in HF with preserved ejection fraction.^[8]^ CHF development can be either directly triggered by immune and inflammatory responses during viral infection or caused by the sustained and chronic detrimental inflammation following myocardial injury.^[11]^ Based on the well-established critical roles of inflammation in CHF, numerous attempts have been made to apply inflammatory factors to develop therapeutic treatment and biomarkers. However, although inflammatory factors and cytokines have been a significant focus on studying CHF for decades, most clinical trials that target inflammatory factors or cytokines in patients with CHF have been negative.^[12]^ Besides, attempts to use inflammatory factors as biomarkers and indicators of CHF have also mainly been negative due to the low specificity and sensitivity, suggesting that the relationship between inflammatory and CHF may be far more complicated. Thus, extensive investigation and new biomarkers are still in demand.

Apart from inflammation, microRNAs (miRNAs) have also been shown to play a critical role in the development of CHF through modulating gene expression.^[13]^ miRNAs are endogenous, non-coding, single-stranded RNAs with a length of around 22 nucleotides.^[14]^ They are generated by processing pre-miRNAs transcribed by RNA polymerase II from the genome.^[15]^ miRNAs modulate gene expression through complementary base-pairing with their specific messenger RNA (mRNA) targets. As a result, those mRNA targets are either cleaved or silenced at the mRNA translational level.^[16]^ A number of miRNAs have been identified to have expression changes during the development of CHF. However, whether these covariances indicate any causation remains unclear.^[17]^ Besides, whether any of the changed miRNAs correlates with CHF severity remains undetermined. miRNA-21 is one of the miRNAs shown to be related to CHF. It was initially identified as a tumor growth enhancer^[18,19]^ and later as mediating the homeostasis of the cardiovascular system.^[20,21]^ Although abnormal levels of miRNA-21 have been found to associate with the development of multiple cardiovascular diseases, including coronary heart disease, cardiac fibrosis, and hypertrophy,^[18,22,23]^ the specific mRNA targets and underlying mechanism have not been determined. Besides, whether changes in serum levels of miRNA-21 during CHF reflect the severity of CHF has not been examined. Furthermore, whether the function of miRNA-21 is related to the well-established inflammatory responses during CHF has not been determined.

In the presented study, we investigated the correlation between serum levels of miRNA-21 and inflammatory factors with respect to the development and severity of CHF, aiming to establish the potential of using the miRNA-21 level to indicate CHF severity and provide new insights into the diagnosis of CHF.

## 2. Materials and Methods

### 2.1. Study population

Retrospectively, the presented study involves a total of 120 (67 male/53 female) adult patients with CHF admitted to the Emergency department of the Second Hospital of Lanzhou University (Lanzhou; Gansu, China) from January 2020 to June 2021 and 100 (58 male/42 female) control health people with non-failing hearts who underwent physical examination during the same time period. All involved patients have met the inclusion criteria and signed an informed consent form for enrollment in the study according to the protocol approved by the Research Ethics Committee of Second Hospital of Lanzhou University. Following enrollment, demographic, clinical history, comorbidity, and laboratory data were collected by reviewing the patient records. Besides, the current study excluded patients with malignant tumors since previous studies indicated that serum miRNA-21 levels increased during malignant tumors.^[24]^

Inclusion criteria: Diagnosis of CHF according to European Society of Cardiology Guidelines for Acute and CHF; Initial diagnosed and hospitalized in the Second Hospital of Lanzhou University; Aged between 18 years old and 80 years old; With normal cognitive function and relevant inspections. Exclusion criteria: With moderate or severe aortic stenosis; With active malignancy, hepatic (cirrhosis or active hepatitis) dysfunction; With moderate or severe rheumatic disease; With concomitant acute coronary syndromes; With a history of heart surgery.

### 2.2. Measurement of serum mir-21 level

Peripheral venous blood specimens were collected in ethylenediaminetetraacetic acid-containing tubes, centrifuged at 3000 r/min for 5 minute to isolate the plasma. Plasma samples were frozen at −80°C until the assays were performed. miRNAs were isolated from 500 µL plasma using mirVana PARIS kit (Ambion; Thermo Fisher Scientific, Inc., Waltham, MA, cat: AM1556) following the manufacturer’s instructions. RNA concentrations were determined by NanoDrop (Thermo Fisher Scientific, Inc., Wilmington, DE, Cat: UX-83061-00). Reverse transcription reactions were performed on 100 ng RNA following the manufacturer’s instructions of TaqMan® miR RT kit (Life Technologies; Thermo Fisher Scientific, Inc. cat: 4366596). Relative serum levels of miRNA-21 were determined through real-time quantitative polymerase chain reaction on the 7500 Real-Time PCR System (Life Technologies; Thermo Fisher Scientific, Inc. cat: 4351105). The PCR reactions were performed at 95°C for 10 minutes followed by 40 cycles of amplification (95°C for 15 second and 60°C for 60 second) with technical triplicates. Relative expression levels for miRNA were estimated using the 2^−ΔΔCt^ method normalized against spike-in UniSp6.

miRNA-21 forward primer: 5’-AGCTGGATGCTGGCATGAT; miRNA-21 reverse primer: 5’-CTGTAAGCTGAAGTCGAAG; UniSp6 forward primer: 5’-CGCTTCGGCAGCACATATAC; UniSp6 Reverse primer: 5’-AAATATGGAACGCTTCACGA.

### 2.3. Measurement of cardiac neurohormone and inflammatory factors

Levels of human brain natriuretic peptide (BNP) were measured by BNP enzyme-linked immunosorbent assay kit (Catalog #: D711347; Sangon; Detection Range: 31.25--2000 pg/mL; Intra-assay CV% < 6%; Inter-assay CV% < 6%). NT-proBNP (Brain natriuretic peptide n-terminal prohormone) were measured by NT-proBNP Rapid test kit (Catalog #: 5194C4X050; dochekbio; Detection Range: ≤100 pg/mL; Intra-assay CV% < 10%; Inter-assay CV% < 15%) using the Fluorescence immunoassay POC analyzers (MedicalExpo, Model: Getein1100).

Inflammatory factors were detected by ELISA kits: hs-CRP (Human hs-CRP ELISA Kit; Catalog #: D711314-0096; Sangon Biotech; Detection Range: 15.63--1000 pg/mL; Intra-assay CV% < 10%; Inter-assay CV% < 10%); tumor necrosis factor-alpha (TNF-α) (Human TNF-α ELISA Kit; Catalog #: D711045-0096; Sangon Biotech; Detection Range: 7.81--500 pg/mL; Intra-assay CV% < 10%; Inter-assay CV% < 10%); IL-6 (Human IL-6(Interleukin 6) ELISA Kit; Catalog #: D711013-0096; Sangon Biotech; Detection Range: 7.81--500 pg/mL; Intra-assay CV% < 10%; Inter-assay CV% < 10%); IL-17 (Human IL-17A(Interleukin 17A) ELISA Kit; Catalog #: D711337-0096; Sangon Biotech; Detection Range: 7.81--500 pg/mL; Intra-assay CV% < 10%; Inter-assay CV% <10%). All the measurements were performed according to the manufacturer’s instructions with technical triplicates. Average values were calculated.

### 2.4. Echocardiography

Vivid7 full-digital color Doppler ultrasound diagnostic apparatus (GE, USA) was used to detect the cardiac function parameters, including the LVEF, right ventricular ejection fraction (RVEF), stroke volume (SV), cardiac index (CI). The New York Society of Cardiology functional classification^[25]^ was used to assess the overall cardiac function status of the study subjects into 4 grades (I–IV), of which grades III and IV indicate severe heart dysfunction. Real-time 3-dimensional echocardiography was used to detect cardiac muscle restructuring parameters, including left ventricular posterior wall (LVPW), left ventricular mass index (LVMI), and left ventricular remodeling index (LVRI).

### 2.5. Statistical analysis

Data were analyzed by SPSS 20.0 statistical software (version 20.0, SPSS, Inc., Chicago, IL). Mapping analysis was performed using Graph Pad Prism 7. Count data were shown with numbers and percentages. Normal distribution of data was performed as ($\bar{x}\pm s$). Student *t* test and Rank Sum Test were performed. Person correlation coefficients were used to assess the correlation among miRNA-21, RVEF, LVEF, SV, CI, TNF-α, hs-CRP, IL-6, and IL-17. Spearman correlation coefficients were used to assess the correlations between miR-21 and HF classification. *P* < .05 was considered to show a statistically significant difference.

## 3. Results

### 3.1. Characteristics of the study population

The baseline clinical characteristics of the study population are summarized in Table 1. Briefly, there is no statistically significant difference (*P* < .05) in gender, body mass index, or diastolic blood pressure between the CHF patients and control patients with non-failing hearts. In contrast, systolic blood pressure and obesity rate increased significantly (*P* < .05) in CHF patients.

**Table 1:** Clinical characteristics and perioperative data.

Parameters	Control (n = 100)	Patients with CHF (n = 120)	*χ*^2^/*t*	*P* value
Age (years)	63.25 ± 8.89	63.10 ± 9.02	0.205	.458
Gender (M/F)	58/42	67/53	0.010	.935
BMI (kg/m^2^)	23.86 ± 1.98	24.01 ± 2.05	0.128	.44
SBP (mm Hg)	121.8 ± 7.9	133.5 ± 18.6	3.58	.01
DBP (mm Hg)	79.6 ± 6.5	80.1 ± 14.3	0.92	.83
Obesity (%)	12.1	40.3	6.71	.01

Note: Data are shown as means ± SD.

*χ^2^/t*, results of chi-square test; BMI, body mass index; CHF = chronic heart failure, DBP, diastolic blood pressure; F: female; M: male; SBP, systolic blood pressure.

*P* value, result of Student *t* test. *P* < .05 is considered to be significant.

### 3.2. CHF patients showed decreased cardiac function and increased myocardial remodeling

**Figure 1.**
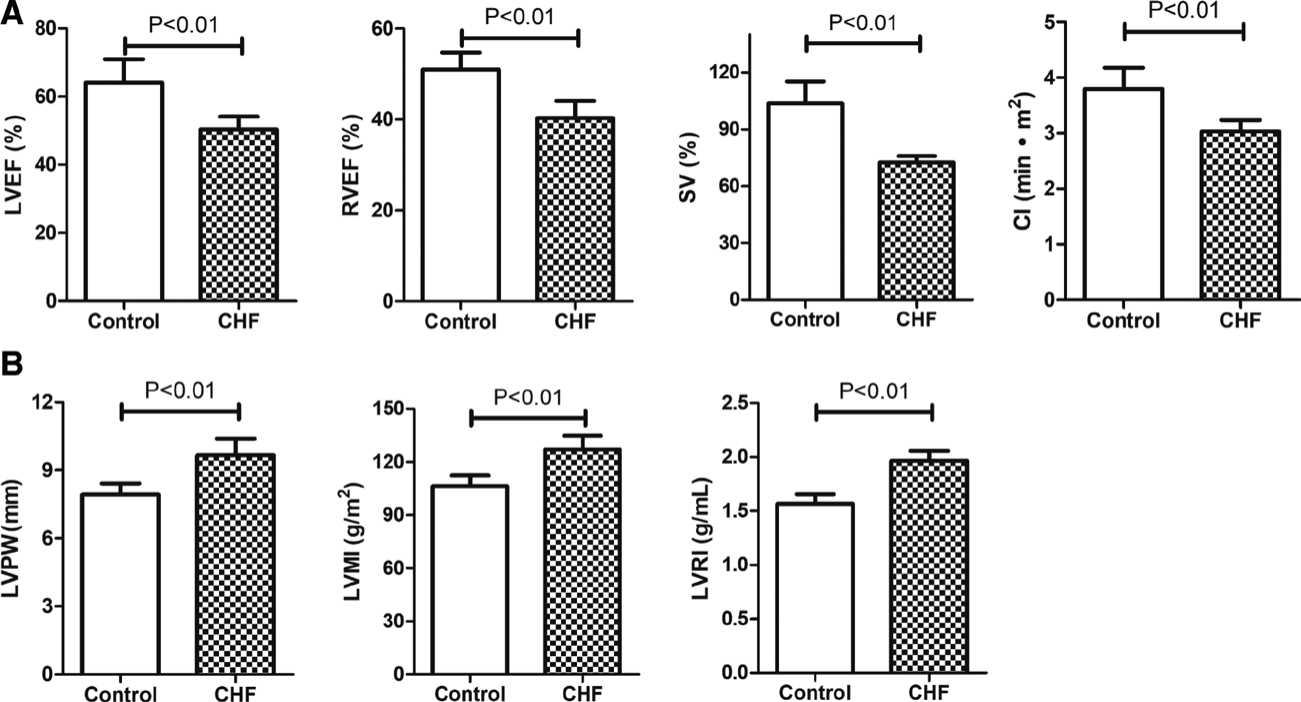
Decreased cardiac function and increased myocardial remodeling in CHF patients. (A) Barplots showing that cardiac function parameters (LVEF, RVEF, SV, CI) decreased significantly in CHF patients. (B) Barplots showing that myocardial remodeling parameters (LVPW, LVMI, and LVRI) increased significantly in CHF patients. *P* < .05 is considered to be significant. CHF = chronic heart failure, CI = cardiac index, LVEF = left ventricular ejection fraction, LVMI = left ventricular mass index, LVPW = left ventricular posterior wall, LVRI =left ventricular remodeling index, RVEF = right ventricular ejection fraction, SV = stroke volume.

To begin with, we summarized the echocardiography results of the involved CHF patients and control patients with non-failing hearts (Fig. 1). As expected, LVEF, RVEF, SV, and CI decreased statistically significantly (*P* < .01) in CHF patients, suggesting decreased cardiac function. In contrast, Real-time 3-dimensional echocardiography showed that LVPW, LVMI, and LVRI increased significantly (*P* < .01) in CHF patients.. We then sub-clustered the CHF patients into 4 groups (I–IV) based on the severity of heart dysfunction (IV is the most severe) according to the New York Society of Cardiology functional classification (Table 2).

**Table 2:** New York Society of Cardiology (NY-HA) functional classification.

Parameters	Class I (n = 27)	Class II (n = 51)	Class III (n = 26)	Class IV (n = 16)	*χ*^2^/*U*	*P* value
Age (years)	62.13 ± 7.54	63.30 ± 8.05	62.28 ± 9.12	63.35 ± 9.46	0.413	.528
Gender (M/F)	14/13	28/23	15/11	9/5	0.013	.926
BMI (kg/m^2^)	22.45 ± 2.38	23.57 ± 1.98	24.05 ± 2.19	23.96 ± 2.16	0.128	.44
SBP (mm Hg)	128.5 ± 15.4	127.8 ± 14.9	105.6 ± 17.9	99.7 ± 10.8	5.49	.01
DBP (mm Hg)	83.4 ± 7.9	78.5 ± 11.8	69.5 ± 10.5	65.8 ± 8.7	6.52	.01
Obesity (%)	15.1	28.9	38.5	45.5	5.47	.027

Note: Data are shown as means ± SD.

*χ^2^/U*, results of chi-square test; BMI, body mass index; DBP, diastolic blood pressure; F: female; M: male; SBP, systolic blood pressure.

*P* value, result of *U* test. *P* < .05 is considered to be significant.

### 3.3. CHF patients showed decreased serum miRNA-21 while increased cardiac neurohormone and inflammatory factors

**Figure 2.**
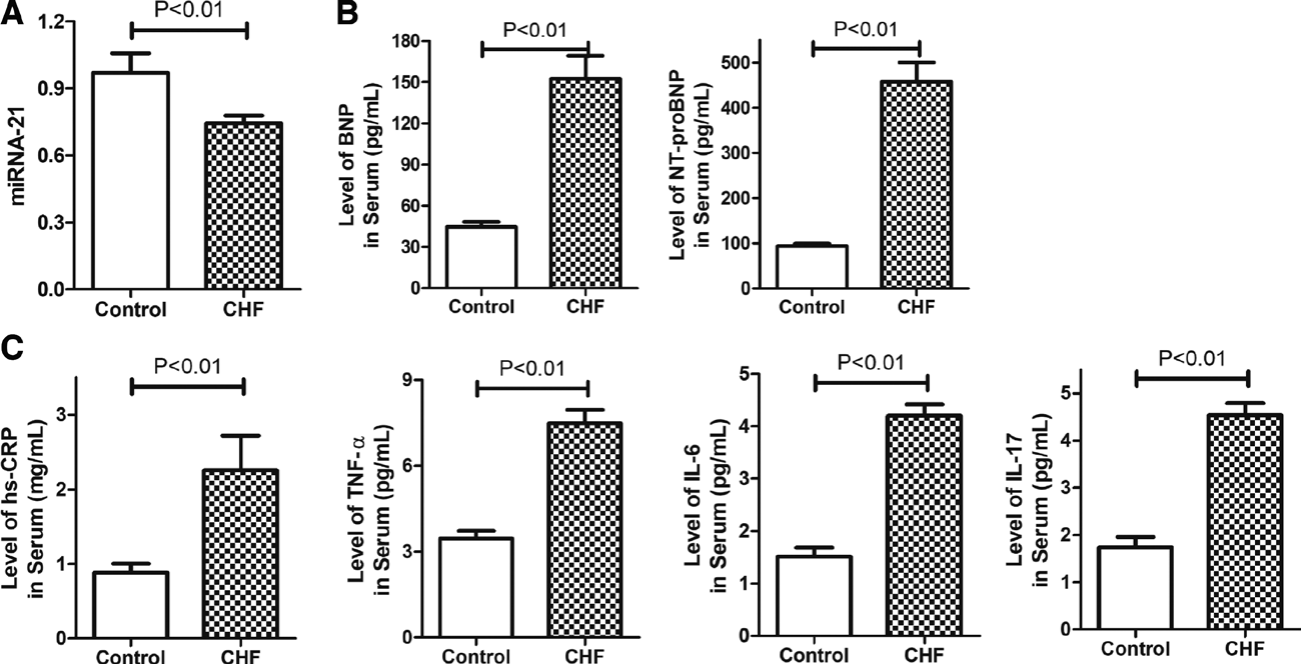
Decreased miRNA-21 and increased cardiac neurohormone and inflammation in CHF patients. (A) Barplot showing miRNA-21 decreased significantly in the peripheral blood in CHF patients. (B) Barplots showing cardiac neurohormone (BNP, NT-proBNP) increased significantly in CHF patients. (C) Barplots showing inflammatory factors (hs-CRP, TNF-α, IL-6, and IL-17) increased significantly in CHF patients. *P* < .05 is considered to be significant. BNP = brain natriuretic peptide, CHF = chronic heart failure, hs-CRP = human hs-CRP, IL-6 = interleukin 6, IL-17 = interleukin 17, miRNAs = microRNAs, NT-proBNP = Brain natriuretic peptide n-terminal prohormone, TNF-α = tumor necrosis factor-alpha.

We next measured miRNA-21, cardiac neurohormone, and inflammatory factors in the peripheral blood of CHF patients and control patients. Similar as have been shown in previous publications, neurohormone (BNP, NT-proBNP) and inflammatory factors (hs-CRP, TNF-α, IL-6, and IL-17) increased significantly (*P* < .05) in CHF patients, confirming the involvement of inflammation in the development of CHF (Fig. 2B and C). On the other hand, miRNA-21 significantly (*P* < .05) decreased in CHF patients compared with control patients (Fig. 2A), suggesting its relevance to HF.

### 3.4. miRNA-21 negatively correlated with cardiac function

**Figure 3.**
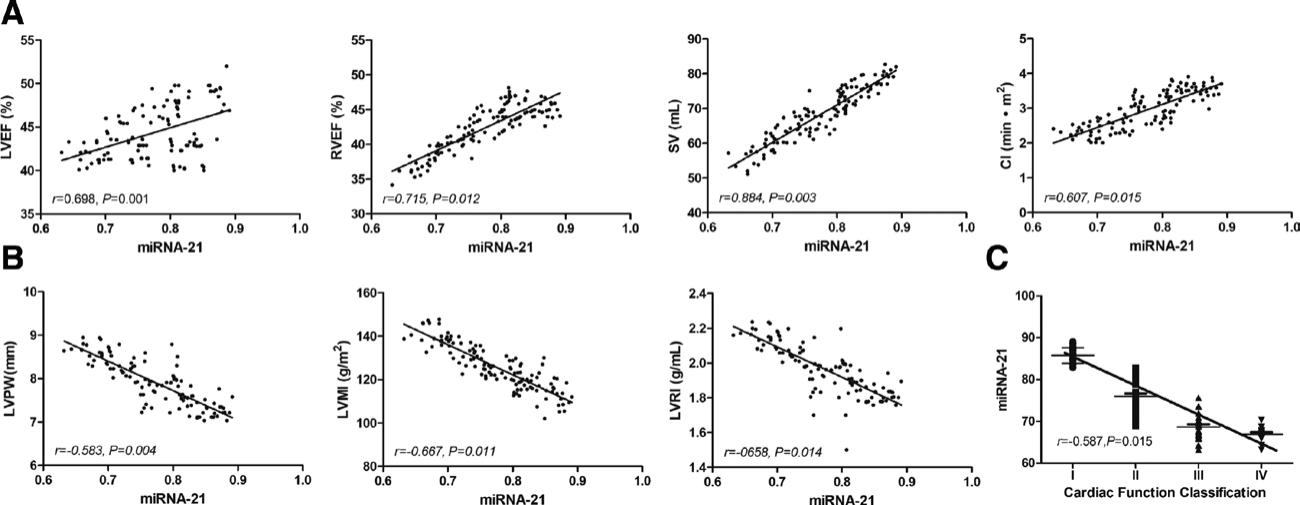
Correlation between miRNA-21 and cardiac function. (A) Dot plots showing the positive correlations between miRNA-21 and cardiac function parameters (LVEF, RVEF, SV, CI). (B) Dot plots showing the negative correlations between miRNA-21 and myocardial remodeling parameters (LVPW, LVMI, LVRI). (C) Dot plot showing that miRNA-21 level decreases from grade I to IV patients. CI = cardiac index, LVEF = left ventricular ejection fraction, LVMI = left ventricular mass index, LVPW = left ventricular posterior wall, LVRI =left ventricular remodeling index, miRNAs = microRNAs, RVEF = right ventricular ejection fraction, SV = stroke volume.

After we observed decreased miRNA-21 in CHF patients, we examined whether these changes in miRNA-21 correlate with cardiac function changes. We found that miRNA-21 levels negatively correlated with cardiac function parameters (LVEF, RVEF, SV, CI) in CHF patients while positively correlated with myocardial remodeling parameters (LVPW, LVMI, LVRI), indicating the linkage between low miRNA-21 level and cardiac dysfunction (Fig. 3A and B). Besides, serum miRNA-21 levels decreased from grade I to IV CHF patients, suggesting miRNA-21 negatively correlated with the severity of CHF (Fig. 3C).

### 3.5. miRNA-21 negatively correlated with inflammation in CHF patients

**Figure 4.**
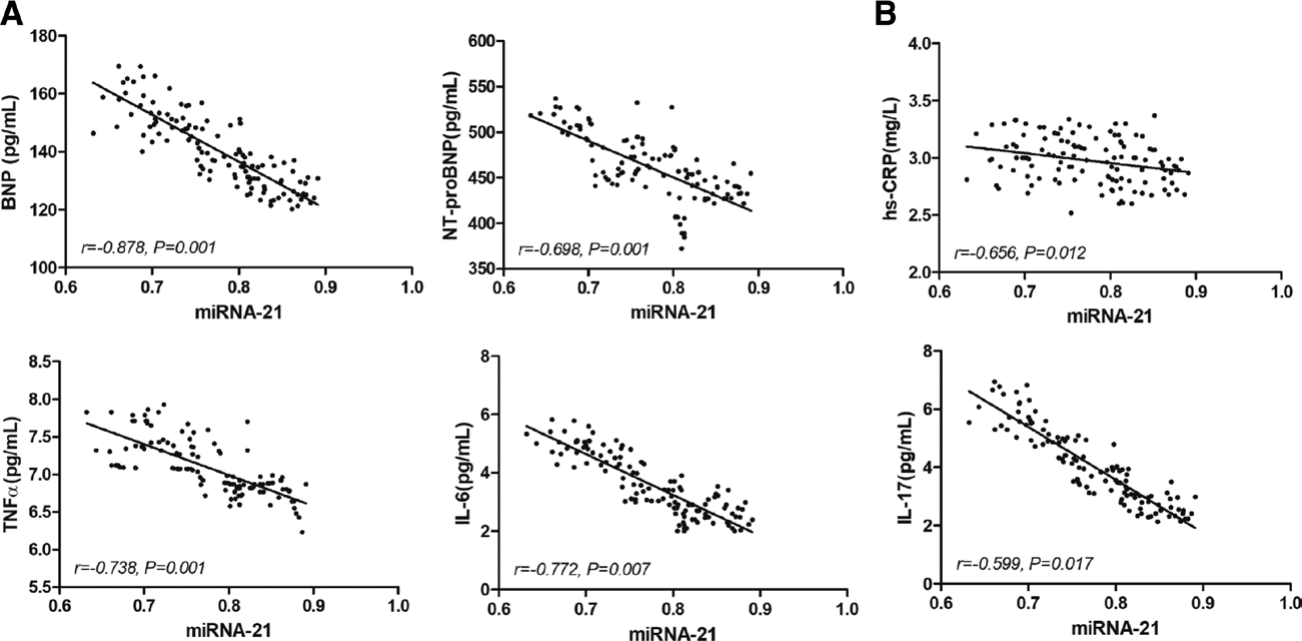
Correlations between miRNA-21 and inflammatory factors. (A) Dot plots showing the correlations between miRNA-21 and cardiac neurohormone. (B) Dot plots showing the correlations between miRNA-21 and inflammatory factors. miRNAs = microRNAs.

Besides the correlations between miRNA-21 and cardiac function, we also observed strong negative correlations between levels of miRNA-21 and levels of cardiac neurohormone and inflammatory factors (Fig. 4), indicating a potential linkage between inflammation and miRNA-21. Furthermore, KEGG (Kyoto Encyclopedia of Genes and Genomes) enrichment analysis on the predicted mRNA targets of miRNA-21 showed the enrichment of inflammation and immune-related pathways such as JAK-STAT signaling and IL12/27 mediated pathway, strengthening the linkage between miRNA-21 and inflammation (Table 3).

**Table 3:** Top enriched KEGG pathways of the predicted mRNA targets of miRNA-21.

Signal pathways	Enrichment *P* value
TGF-beta signaling pathway	7.52E-07
JAK-STAT signaling pathway	3.07E-05
L27-mediated signaling events	6.49E-04
Innate immune system	2.17E-03
IL12-mediated signaling events	3.09E-03

*Note*: Targets of miRNA-21 were predicted through miR System database. KEGG enriched pathways are ordered based on enrichment *P* values. Top 5 pathways are shown.

IL-12 = interleukin 12; KEGG = Kyoto Encyclopedia of Genes and Genomes, miRNAs = microRNAs, mRNA = messenger RNA.

## 4. Discussion

In the presented study, we investigated changes of miRNA-21 during the development of CHF and its correlation with inflammation and the severity of CHF. We observed significantly decreased levels of miRNA-21 and increased levels of inflammation factors in peripheral blood of CHF patients (*P* < .05). Levels of miRNA-21 became even lower as CHF became severer, correlating with decreased cardiac function and increased myocardial remodeling, which suggested the potential of using miRNA-21 to indicate the severity of CHF during CHF diagnosis. Besides, we also observed a strong negative correlation between miRNA-21 and inflammatory factors. Considering that the predicted mRNA targets of miRNA-21 are enriched in inflammatory-related pathways, miRNA-21 might function as regulating inflammation in the pathogenesis of CHF.

miRNAs are small non-coding regulatory RNAs transcribed by RNA polymerase II.^[14]^ miRNAs modulate gene expression through complementary basing to their target mRNA, which will either cleave the target mRNAs or cause translation repression of the targets.^[26]^ Depending on the function of different targets, miRNAs can be involved in various biological, physiological, and pathological processes.^[27]^ miRNA-21 is one of the miRNAs that have been found related to human diseases. It is initially identified as a tumor growth enhancer^[24,28]^ and related to right ventricular dysfunction and hypertension.^[29]^ In the presented study, we observed decreased levels of miRNA-21 in peripheral blood of the CHF patients, suggesting the cardiac protective function of miRNA-21. The decreased levels of miRNA-21 positively correlate with decreased cardiac function, which agrees with the previous studies showing miRNA-21 protects cardiomyocytes against Ischemia/reperfusion.^[30–32]^ Besides, miRNA-21 level negatively correlates with myocardial remodeling, consistent with the previous studies showing decreased angiogenesis and increased myocardial damage upon depletion of miRNA-21.^[30–32]^ Although contradicting results showing increased miR-21 level in patients with hypertension-induced heart disease has been reported previously by Watanabe, et al,^[33]^ the diversity and complicity of heart disease etiology indicates the possibility that miRNA-21 plays different or even opposing roles related to heart disease types. In fact, HF cases involved in our study could hardly be hypertension-induced since only minor increase in systolic blood pressure was observed while no significant difference in diastolic blood pressure observed. Altogether, given the observed correlation between miRNA-21 and the severity of CHF, we suggest the potential to use miRNA-21 levels as an indicator of CHF severity during CHF diagnosis.

Although the molecular mechanism of how miRNA-21 is related to CHF is unclear, it is highly possible that targets of miRNA-21 may be genes promoting CHF based on the decreased levels of miRNA-21 in CHF and the biological function of miRNAs. Therefore, decreased miRNA-21 causes increased expression of its target, resulting in HF. Our KEGG enrichment results showed that the predicted targets of miRNA-21 significantly enriched in inflammation and immune-related pathways, suggesting the possibility that miRNA-21 regulates inflammation through modulating the expression of critical genes involved in inflammatory responses. As inflammation has been identified as a critical driver in different types of CHF, which promotes myocardial remodeling and injury,^[8]^ it is possible that miRNA-21 functions through inflammation during the development of CHF. To study this, we examined the changes of 4 critical inflammatory factors (hs-CRP, TNF-α, IL-6, and IL-17) in CHF and calculated the correlation between these inflammatory factors and miRNA-21. hs-CRP is an inflammatory factor sensitively reflecting the severity of the inflammatory reaction and promoting the activation of TNF-α and IL-6.^[34]^ IL-17 is secreted by Th17 cells and has a solid pro-inflammatory effect promoting the recruitment of monocytes and macrophages.^[35]^ In contrast to miRNA-21, all 4 inflammatory factors significantly increase in CHF patients, confirming the well-evident involvement of inflammation in CHF. Besides, strong negative correlations between the measured inflammatory factors and miRNA-21 were detected even though none of them were predicted as miRNA-21 targets, supporting the idea that miRNA-21 functions through inflammation. However, targets of miRNA-21 and how they are related to inflammation are more complicated and require further extensive studies and experimental validation.

As the leading cause of hospitalization and mortality worldwide, HF, especially CHF, has caused a significant burden on the patients and their families. Although significant improvements have been made in medical care and health services, the steadily increasing prevalence and incidence of CHF still emphasize the high demand for rapid, accurate diagnosis and effective treatment of CHF. Assessment of biomarker concentrations in blood is a quick, noninvasive and practical diagnostic method. For the last decades, multiple biomarkers such as inflammatory factors have been identified to change significantly during CHF. However, most of them are either not sensitive enough or not specific enough to indicate the severity of CHF. Our study suggests the potential of using miRNA-21 as a diagnostic biomarker and indicator of CHF severity based on its high correlation with the severity of CHF, providing insights into the development of diagnostic approaches. Apart from diagnosis, therapeutic targets for effective treatment of CHF is also in demand since most of the CHF clinical trials (mainly focusing on targeting inflammatory factor) have been negative. Our study also suggests the potential cardiac protective function of miRNA-21, provides ideas that RNA-based treatment may be developed for CHF based on miRNA-21.

Although our promising study provides new ideas about the diagnosis and treatment of CHF, certain limitations cannot be ignored. Our study is based on retrospective data collected from the Lanzhou area in the northwest of China, which may not be enough to represent a global trend. Cases involved have an age range varying from 18 to 80, thus different etiologies of HF are probably included. Targets of miRNA-21 are purely predicted in our study and require further experimental validation. Different subtypes of CHF (HF with reduced ejection fraction and HEpEF) were not separated in this study. Further studies are in demand to identify potential biomarkers or understand the pathogenesis of CHF.

## 5. Conclusion

In summary, our study demonstrated the negative correlation between miRNA-21 and the severity of CHF, suggesting the potential of using miRNA as a diagnostic biomarker and indicator of CHF severity. Besides, we demonstrated a strong negative correlation between miRNA-21 and inflammation, indicating miRNA-21 may function through inflammation during the development of CHF, suggesting the potential of the development of miRNA-based treatment of CHF.

## Author contributions

**Conceptualization**: Weiwei Li, Yanan Li.

**Data curation**: Weiwei Li, Yanan Li.

**Formal analysis:** Huan Liu.

**Funding acquisition:** Weiwei Li.

**Investigation**: Weiwei Li, Yanan Li.

**Methodology**: Weiwei Li, Yanan Li.

**Project administration**: Weiwei Li.

**Resources**: Weiwei Li, Yanan Li.

**Supervision**: Weiwei Li.

**Writing—original draft**: Weiwei Li, Feng Jiang, Huan Liu.

**Writing—review & editing**: Weiwei Li, Feng Jiang, Huan Liu.
